# Expert Consensus on the Clinical Application of PI3K/AKT/mTOR Inhibitors in the Treatment of Breast Cancer (2025 Edition)

**DOI:** 10.1002/cai2.70008

**Published:** 2025-04-09

**Authors:** The Breast Cancer Expert Committee of the National Quality Control Center for Cancer, The Expert Committee on Cancer Prevention and Treatment of the Health China Research Center, The Society of Clinical Research on Oncology Medications of the China Anti‐Cancer Association, The Society of Onco‐Pathology of the China Anti‐Cancer Association

**Keywords:** AKT inhibitor, breast cancer, expert consensus, hormone receptor, mTOR inhibitor, PI3K inhibitor

## Abstract

**Background:**

The phosphoinositide 3‐kinase (PI3K)/protein kinase B (PKB or AKT)/mammalian target of rapamycin (mTOR) signaling pathway (PAM pathway) plays a critical role in breast cancer pathogenesis and progression, and is closely linked with resistance to endocrine therapy in advanced breast cancer. Randomized clinical trials have shown that PI3K/AKT/mTOR inhibitors deliver significant clinical benefits, particularly for patients with advanced hormone receptor (HR)‐positive, human epidermal growth factor receptor 2 (HER2)‐negative breast cancer.

**Methods:**

In 2022, the Breast Cancer Expert Committee of the National Cancer Quality Control Center convened specialists in related fields to draft the “Expert Consensus on the Clinical Application of PI3K/AKT/mTOR Inhibitors in the Treatment of Advanced Breast Cancer.” This consensus raised awareness of these inhibitors among oncologists in China and improved the precision of clinical decision‐making. In recent years, growing evidence has emphasized the importance of targeting the PAM pathway, reflected in the approval of several innovative agents. This consensus is an updated 2025 edition that retains the foundational structure of the 2022 edition while incorporating notable updates.

**Results:**

Updates to the consensus include the introduction of newly approved PAM pathway inhibitors, updated data from recent clinical trials, and expanded therapeutic applications. The revised guidance also offers updated recommendations for genetic testing to detect alterations in relevant pathways. The section on managing drug‐related adverse events has been significantly expanded, providing detailed insights into different types of adverse events and their management. These updates aim to enhance the clinical application of PAM pathway inhibitors, promote precision medicine, and ultimately, improve survival outcomes for patients with breast cancer.

**Abbreviations**
AI: aromatase inhibitor
BCRP: breast cancer resistance protein
BSA: body surface area
CDK4/6: dysregulation of cyclin D‐cyclin‐dependent kinase 4/6
CYP2C9: cytochrome P450 2C9
CYP3A4: Cytochrome P450 3A4
ER: estrogen receptor
FDA: Food and Drug Administration
FPG: fasting plasma glucose
HbA1c: glycosylated hemoglobin
HER2: human epidermal growth factor receptor 2
HR: hormone receptor
IHC: immunohistochemistry
NCCN: National Comprehensive Cancer Network
NGS: next‐generation sequencing
NMPA: National Medical Products Administration
OS: overall survival
P‐gp: P‐glycoprotein
p70S6K: p70 ribosomal protein S6 kinase
PFS: progression‐free survival
PI3K/AKT/mTOR: phosphoinositide 3‐kinase/protein kinase B/mammalian target of rapamycin
PIK3CA: phosphatidylinositol‐4,5‐bisphosphate 3‐kinase
PIP2: phosphatidylinositol 4,5‐bisphosphate
PIP3: phosphatidylinositol‐3,4,5‐triphosphate
PTEN: phosphatase and tensin homolog deleted on chromosome 10
RT‐PCR: reverse transcription polymerase chain reaction
RTK: receptor tyrosine kinase
ULN: upper limit of normal

## 1 Introduction

Breast cancer is the most common malignant tumor in women globally in terms of incidence and holds the second‐highest mortality rate among this population [1]. Approximately 70% of breast cancer cases are hormone receptor (HR)‐positive [2, 3]. Treatments such as endocrine therapy, chemotherapy, and targeted approaches, including CDK4/6 inhibitors (CDK4/6i) and anti‐human epidermal growth factor receptor 2 (HER2) therapies, have notably reduced the recurrence rates of this subtype. However, the majority of patients eventually develop drug resistance, resulting in disease progression [4, 5]. This underscores the urgent need to investigate the mechanisms underlying drug resistance and to identify effective strategies to overcome this challenge.

The phosphoinositide 3‐kinase (PI3K)/protein kinase B (PKB or AKT)/mammalian target of rapamycin (mTOR) signaling pathway (commonly referred to as the PAM signaling pathway) plays a critical role in regulating tumor cell growth, proliferation, survival, angiogenesis, and related processes. This pathway is a key contributor to the pathogenesis and progression of breast cancer [5, 6, 7, 8, 9]. Notably, overactivation of the PAM pathway represents one of the major mechanisms of the resistance of endocrine therapy, chemotherapy, and targeted therapy. Based on the specific gene loci targeted within the PAM pathway, its inhibitors are categorized into three primary classes: PI3K inhibitors, AKT inhibitors, and mTOR inhibitors [4, 10, 11]. Among these, the mTOR inhibitor, Everolimus, has been approved by the US Food and Drug Administration (FDA) and the National Medical Products Administration (NMPA) for use combined with exemestane. This combination is indicated for postmenopausal women with HR‐positive/HER2‐negative advanced breast cancer who have experienced treatment failure with letrozole or anastrozole therapy [12]. The PI3Kα inhibitor Inavolisib has also received FDA and NMPA approval. Inavolisib is approved for use in combination with palbociclib and fulvestrant for the treatment of endocrine‐resistant, *PIK3CA*‐mutated HR‐positive/HER2‐negative locally advanced or metastatic breast cancer patients following recurrence during or after completing adjuvant endocrine therapy [13, 16]. The other PI3Kα inhibitor Alpelisib is approved by FDA for use with fulvestrant to treat male and postmenopausal female patients with *PIK3CA*‐mutated, HR‐positive/HER2‐negative advanced breast cancer that has progressed during or after endocrine therapy [17]. Similarly, the AKT inhibitor capivasertib has been approved by the FDA for use in combination with fulvestrant. This combination is suggested for patients with HR‐positive/HER2‐negative, locally advanced, or metastatic breast cancer who exhibit one or more *PIK3CA/AKT1/PTEN* alterations and have progressed on at least one endocrine‐based regimen in the metastatic setting or within 12 months of completing adjuvant therapy [18].

The Society of Clinical Research on Oncology Medications of the China Anti‐Cancer Association, in collaboration with the Breast Cancer Expert Committee of the National Cancer Quality Control Center, the Society of Onco‐Pathology of the China Anti‐Cancer Association, and the Boao Cancer Innovation Institute, convened experts from relevant fields to develop the “Expert Consensus on the Clinical Application of PI3K/AKT/mTOR Inhibitors in the Treatment of Advanced Breast Cancer” in 2022 [19]. To further standardize the clinical application and improve the safety management of PAM‐targeted therapies, the expert panel has since updated the consensus. This revised document provides a comprehensive summary of PAM pathway inhibitors currently available both domestically and internationally. It details their mechanisms of action, clinical efficacy, adverse event management, and critical aspects of related gene mutation testing. The updates aim to support the effective clinical use of these drugs, facilitate efficacy monitoring, and enhance the management of associated adverse events.

## 2 The PAM Pathway Mediates the Pathogenesis, Progression, and Drug Resistance of Breast Cancer

### 2.1 Role of the PAM Pathway in the Pathogenesis and Progression of Breast Cancer

The PAM signaling pathway is characterized predominantly by the induction of receptor tyrosine kinases (RTKs), which trigger the activation of the PI3K complex (comprising p85 and p110 subunits). The PI3K complex converts phosphatidylinositol 4,5‐bisphosphate (PIP2) into phosphatidylinositol 3,4,5‐trisphosphate (PIP3). PIP3 subsequently recruits AKT proteins, prompting their translocation to the cell membrane, where they undergo phosphorylation and activation. Once activated, AKT kinase regulates over a hundred downstream substrates, including the mTOR complex [7, 8, 10, 20, 21]. The phosphatase and tensin homolog (PTEN), encoded by a gene located on human chromosome 10, plays a crucial regulatory role by preventing AKT activation and its downstream effects. This is achieved through the dephosphorylation of PIP3 back to PIP2. Furthermore, PTEN can dephosphorylate various protein substrates that are linked to specific cellular functions, including inhibiting migration, inducing cell cycle arrest, and suppressing stem cell self‐renewal [22, 23, 24]. The PAM pathway is the most commonly activated signaling pathway in breast cancer, which may result from activating mutations in the *PIK3CA* and *AKT1* genes or inactivating alterations in the *PTEN* gene. These alterations regulate critical cellular processes such as tumor initiation, growth, proliferation, migration, invasion, resistance to apoptosis, and angiogenesis [6, 7, 8, 9].

The PI3K complex operates upstream of the PAM pathway and consists of three classes, with Class I being the primary driver of tumor pathogenesis [25]. Research has shown that gain‐of‐function mutations in *PIK3CA* (the gene encoding p110α, the catalytic subunit) are linked to the overactivation of PI3K, a key mechanism in oncogenesis, especially in breast cancer [4, 25]. Additionally, AKT, one of the primary effector molecules downstream of PI3K, is categorized into three subtypes. AKT activation in tumors arises from upstream signaling pathway activation, *AKT1* mutations, loss of *PTEN* function, and *PIK3CA* mutations. Once activated, AKT phosphorylates numerous target molecules within the nucleus and cytosol, triggering a cascade that activates a wide range of downstream substrates. These substrates regulate processes such as cell proliferation, thereby driving tumor cell growth, proliferation, and angiogenesis [6, 20, 26].

Furthermore, AKT promotes the survival and inhibits apoptosis of tumor cells by mechanisms such as inhibiting the pro‐apoptotic Bcl‐2 family members BAD and BAX and negatively regulating forkhead transcription factors like FOXO.

A significant downstream target of the PI3K/AKT pathway is mTOR, whose activation leads to the phosphorylation of ribosomal protein S6 kinase (S6K) and eukaryotic translation initiation factor 4E (eIF4E)‐binding protein 1 (4EBP‐1), which facilitates the transcription, proliferation, growth, and protein synthesis of tumor cells [6, 8, 21, 25, 27].

### 2.2 Epidemiology of PAM Signaling Pathway Alterations in Patients With Breast Cancer

Clinical studies on breast cancer have consistently demonstrated a relatively high prevalence of *PIK3CA* gene mutations among patients with breast cancer. Additionally, *AKT1* point mutations and *PTEN* functional inactivation alterations have been identified across diverse population data sets. In the Chinese population, the carrying rate of *PIK3CA* mutations ranges from 32% to 46.5% [28, 29, 30, 31], markedly higher than corresponding data for Western populations recorded in The Cancer Genome Atlas (TCGA) database (45.6% vs. 34.7%) [29]. Within various breast cancer subtypes, the distribution of *PIK3CA* mutations shows slight variability, peaking at 49.5% in luminal A subtypes and dropping to 16.1% in basal‐like breast cancer [28]. Similarly, the mutation rate of AKT1 in the Chinese population is significantly elevated compared to the Western population (6.4% vs. 2.5% in TCGA). Among luminal A tumors, the occurrence rate of *AKT1* mutations reaches 12.1% [28]. Notably, the E17K activating mutation, located in the PH domain of *AKT1*, is a hotspot mutation in the Chinese breast cancer population, with a carrier rate of 3.1% [32], and accounting for 90.9% of all *AKT1* mutations in luminal A subtypes [28]. The mechanisms underlying *PTEN* gene mutations are diverse and include gene mutations, large fragment variations (e.g., copy number loss and genomic rearrangements), and abnormalities in regulatory and epigenetic modifications affecting transcription and translation processes [33]. The mutation frequency of *PTEN* in the Chinese breast cancer population is 3.7%, closely aligning with data for white individuals in the TCGA database (4.6%) [28]. Analysis of *PTEN* copy number alterations reveals differences in deletion rates across breast cancer subtypes, with basal‐like breast cancer samples exhibiting the highest deletion rate of 9.7% and a total loss rate of 36.6% [28]. Moreover, *PTEN* gene inactivation mutations can coexist with *PIK3CA* activating mutations in 1.3% of cases [34].

### 2.3 The Role of the PAM Signaling Pathway in the Resistance to Breast Cancer Treatment

#### 2.3.1 Resistance to Endocrine Therapy

A potential mechanism of resistance to endocrine therapy involves overactivation of the PAM pathway [35]. Within this pathway, *PIK3CA* is the most frequently mutated gene, identified in nearly 50% of patients with estrogen receptor (ER)‐positive breast cancer [4]. Preclinical studies have demonstrated that PI3K and AKT can phosphorylate the Ser167 locus of ERα, leading to independent activation of ERα even in the absence of estrogen. This interaction between the ER and overactivation of the PAM pathway allows breast cancer cells to adapt to estrogen deprivation, ultimately rendering them less sensitive to endocrine therapy [4, 6]. During the progression of endocrine resistance, following long‐term estrogen deprivation, PI3K primarily activates the mTOR substrate p70 ribosomal protein S6 kinase (p70S6K) in the human breast cancer cell lines MCF‐7 and MDA‐MB‐361 [4]. Additionally, *PIK3CA* and *AKT1* mutations, as well as the loss of *PTEN*, contribute to the estrogen‐independent growth of breast cancer cells. Furthermore, the upregulation of RTKs, which enhances activation of the downstream PAM pathway, also plays a significant role in the mechanism of resistance [4].

#### 2.3.2 Resistance to CDK4/6i

The PAM pathway also plays a role in the development of acquired resistance to CDK4/6i. Among the key mechanisms contributing to drug resistance against CDK4/6iin HR‐positive/HER2‐negative breast cancer cells, upregulation of the PI3K/mTOR pathway is particularly common, making it a promising target for overcoming resistance [5, 36]. An exploratory biomarker analysis from the Young Pearl study revealed that loss of *PTEN* and alterations in the retinoblastoma tumor suppressor (RB1) pathway are associated with poor outcomes when palbociclib is combined with endocrine therapy [37]. In a model of acquired resistance to palbociclib monotherapy, resistant cell lines demonstrated significant changes in proteins associated with the PAM pathway. These included upregulation of pAKT^S473^, pAKT^T308^, and P70S6K, as well as downregulation of *PTEN*, compared to their parental counterparts. Pathway enrichment analysis identified several alterations in cancer‐related signaling pathways, such as PI3K, mTOR, AMP‐activated protein kinase (AMPK), and pathways related to apoptosis induction [5]. Research indicates that targeting and inhibiting the PI3K/mTOR pathway can restore tumor cell sensitivity to palbociclib [38]. Furthermore, blocking the PI3K/AKT/mTOR signaling pathway has been shown to effectively suppress the proliferation of palbociclib‐resistant cells [39]. Preclinical studies suggest that simultaneous inhibition of the ER, CDK4/6, and PAM signaling pathways can reverse or delay resistance to CDK4/6i [40].

In tumor specimens obtained after resistance to palbociclib treatment, significant increases were observed in the expression of Cyclin D1, CDK4, p‐AKT, and p‐4E‐BP1. Notably, targeting the PI3K/mTOR pathway significantly reduced the levels of proteins such as Cyclin D1 and CDK4 [38].

#### 2.3.3 Resistance to Anti‐HER2 Therapy

As a member of the RTK ErbB family of proteins, HER2 forms heterodimers with other family members, initiating self‐phosphorylation of its RTK domain. This process activates downstream signaling pathways, including the PAM pathway. Aberrant activation of these pathways can lead to significant dysregulation in cellular processes.

The PAM pathway is closely linked to resistance against anti‐HER2 therapies [4, 41]. The most prevalent variation of the HER2 receptor molecule is its truncated form, p95‐HER2, and overexpression of p95‐HER2 induces resistance to anti‐HER2 therapies by activating the PI3K/AKT signaling pathway [4]. Additionally, trastuzumab, an anti‐HER2 agent, promotes the expression of HER3 in breast cancer cells. The overexpression of HER3 further activates the PI3K/AKT pathway, leading to resistance to anti‐HER2 therapies [4]. Other factors also contribute to the activation of the PAM pathway, intensifying resistance mechanisms.

This pathway, involving the *PIK3CA* mutation, *AKT1* mutation, *AKT2* amplification, and the loss of the tumor suppressor gene *PTEN*, can contribute to resistance against anti‐HER2 agents like trastuzumab and lapatinib [4, 41].

#### 2.3.4 Resistance to Chemotherapy

The transcription of breast cancer resistance protein (BCRP) and the expression of P‐glycoprotein (P‐gp) is regulated through the PAM pathway, influenced by the KEAP1‐Nrf2 axis and the nuclear factor kappa‐B (NF‐κB) pathway, although the precise mechanisms remain unclear. BCRP plays a critical role in enhancing the efflux of chemotherapy agents. P‐gp contributes to the development of chemoresistance [4]. Similarly, the phosphorylation of AKT has been shown to have a positive correlation with the activity, migration, and apoptosis of breast cancer cells, which may further drive chemoresistance [42].

## 3 Clinical Application Guidance

### 3.1 The Efficacy of PAM Pathway Inhibitors

#### 3.1.1 PI3K Inhibitors

##### 3.1.1.1 Inavolisib

The phase III clinical trial, INAVO120, evaluated the efficacy and safety of inavolisib in combination with palbociclib and fulvestrant for patients with *PIK3CA*‐mutated, HR‐positive/HER2‐negative locally advanced or metastatic breast cancer who experienced disease progression within 12 months during or after adjuvant endocrine therapy [43]. The results demonstrated that median progression‐free survival (PFS) was significantly prolonged in the treatment group receiving inavolisib in combination with palbociclib and fulvestrant compared to the placebo group treated with palbociclib and fulvestrant (15.0 vs. 7.3 months, hazard ratio [HR] = 0.43). Consistent benefits were observed across predefined clinical subgroups, including variations in race, geographic region, baseline Eastern Cooperative Oncology Group Performance Status (ECOG PS) scores, menopausal status, liver metastasis status, and number of metastatic sites. Although median overall survival (OS) for the combination therapy group has not yet been reached, a clear trend favoring benefit has emerged. Additionally, the time from randomization to the first requirement for subsequent chemotherapy was significantly extended in the inavolisib treatment group (not reached vs. 15.0 months, HR = 0.54) [44]. This further highlights the sustained advantages of inavolisib‐based therapy, as it delays disease progression and the need for subsequent treatments, including chemotherapy.

Based on findings of the INAVO120 study, the FDA and NMPA has approved the combination of inavolisib, palbociclib, and fulvestrant for the treatment of patients with endocrine‐resistant, *PIK3CA*‐mutated, HR‐positive/HER2‐negative locally advanced or metastatic breast cancer, who experience disease recurrence during or after adjuvant endocrine therapy [13, 16]. Additionally, the latest National Comprehensive Cancer Network (NCCN) Clinical Practice Guidelines for Breast Cancer (2025, Version 2) recommend inavolisib in combination with palbociclib and fulvestrant as a first‐line treatment for patients with advanced *PIK3CA*‐mutated, HR‐positive/HER2‐negative breast cancer that relapse within 12 months during or after adjuvant endocrine therapy (Category I) [45].

##### 3.1.1.2 Alpelisib

For patients with HR‐positive/HER2‐negative advanced breast cancer who previously underwent endocrine therapy, the SOLAR‐1 study assessed the efficacy and safety of combining fulvestrant with alpelisib versus fulvestrant and placebo. Results revealed that, in patients with *PIK3CA* mutations, the combination of alpelisib and fulvestrant significantly prolonged median PFS compared to fulvestrant monotherapy (11.0 vs. 5.7 months; HR = 0.65) [46]. Final OS results further underscored the efficacy of this regimen, showing that median OS in the alpelisib combined with the fulvestrant group was extended by 7.9 months compared with the control group (39.3 vs. 31.4 months) [47]. Additionally, the phase II BYLieve study provided evidence that HR‐positive/HER2‐negative advanced breast cancer patients with *PIK3CA* mutations, who had previously been treated with CDK4/6i, benefited from alpelisib in combination with either aromatase inhibitors (AI) or fulvestrant [48]. Importantly, the duration of prior CDK4/6 inhibitor therapy did not impact the efficacy or safety profile of alpelisib, highlighting it as a viable treatment option for patients exhibiting resistance to CDK4/6i [49].

Based on these research findings, the NCCN Clinical Practice Guidelines for Breast Cancer 2025, Version 2, recommend alpelisib in combination with fulvestrant as the preferred second‐line treatment (Class 1) for postmenopausal breast cancer patients with HR‐positive/HER2‐negative PIK3CA mutations [45]. Similarly, the 6th and 7th Edition European School of Oncology‐European Society for Medical Oncology (ESO‐ESMO) International Consensus Guidelines: Advanced Breast Cancer (ABC6/7) suggest that, for patients previously treated with AIs who are maintaining appropriate glycosylated hemoglobin (HbA1c) levels, alpelisib combined with fulvestrant remains a viable treatment option for tumors with *PIK3CA* mutations in exon 9 or 20 [50].

#### 3.1.2 AKT inhibitors

##### Capivasertib

The CAPItello‐291 (NCT04305496) study is a phase III clinical trial focusing on patients with HR‐positive/HER2‐negative advanced breast cancer previously treated with CDK4/6i [51]. Its objective is to compare the efficacy and safety of capivasertib combined with fulvestrant versus a placebo combined with fulvestrant. The findings revealed that the capivasertib plus fulvestrant group demonstrated a significant improvement in PFS compared to the placebo‐plus‐fulvestrant group. This was observed in both the overall population and the *PIK3CA/AKT1/PTEN*‐altered subgroup, aligning with results from the phase II FAKTION study [52]. Specifically, median PFS in the overall population was 7.2 versus 3.6 months (HR = 0.60) and 7.3 versus 3.1 months (HR = 0.50) in the *PIK3CA/AKT1/PTEN*‐altered subgroup. Additionally, consistent benefits were noted across all predefined clinical subgroups [51].

While OS data have not yet matured, an observable trend suggests potential benefits. Notably, the time from randomization to the second progression and initiation of the first chemotherapy was significantly extended in the capivasertib plus fulvestrant group [53]. This finding highlights the sustained advantages of this combination therapy and its potential to delay the need for chemotherapy for patients.

Based on the findings of the CAPItello‐291 study, the FDA has approved the use of capivasertib in combination with fulvestrant for treating patients with HR‐positive/HER2‐negative locally advanced or metastatic breast cancer harboring one or more *PIK3CA*, *AKT1*, or *PTEN* gene alterations [18]. Both the latest NCCN guidelines [45] and the updated American Society of Clinical Oncology (ASCO) guidelines [54] recommend this combination as the preferred second‐line treatment for advanced‐stage patients with these specific alterations (Category I).

#### 3.1.3 mTOR Inhibitors

##### Everolimus

The phase III BOLERO‐2 study demonstrated that the combination of everolimus and exemestane significantly prolongs PFS compared to exemestane alone in postmenopausal HR‐positive/HER2‐negative advanced breast cancer patients who have experienced AI treatment failure. The median PFS was extended to 7.8 months with the combination therapy, compared to 3.2 months with exemestane alone [55]. Similarly, the phase II BOLERO‐5 study, which evaluated the efficacy of everolimus combined with exemestane in Chinese postmenopausal ER‐positive/HER2‐negative advanced breast cancer patients, produced results consistent with those of the international multicenter BOLERO‐2 study. This study also reported a significant prolongation of median PFS with combination therapy (7.4 vs. 2.0 months), further underscoring the efficacy of everolimus and exemestane in this patient population [56]. In addition, the phase II MIRACLE study, conducted within the Chinese population, confirmed that everolimus combined with endocrine therapy as a first‐line treatment significantly extends PFS in premenopausal HR‐positive/HER2‐negative advanced breast cancer patients with endocrine treatment failure compared to endocrine therapy alone (19.4 vs. 12.9 months) [57]. Subgroup analyses from both the BOLERO‐2 and MIRACLE studies revealed that the PFS benefit of everolimus combined with endocrine therapy remained consistent across various patient subgroups. Factors such as age (< 65 years or ≥ 65 years), the presence of visceral metastases, bone metastases, primary or secondary endocrine resistance, or recurrence during adjuvant therapy did not diminish the observed benefit [55, 57]. Based on the findings of the BOLERO‐2 and BOLERO‐5 studies, everolimus has been approved by the FDA [12] and the NMPA for use in combination with exemestane to treat postmenopausal HR‐positive/HER2‐negative advanced breast cancer patients who have experienced treatment failure after letrozole or anastrozole therapy.

The NCCN guidelines recommend the use of everolimus in combination with endocrine therapy as a second‐line or later treatment for postmenopausal patients with HR‐positive/HER2‐negative advanced breast cancer [45]. Similarly, the ASCO guidelines suggest everolimus combined with fulvestrant as a treatment option for advanced second‐line therapy in HR‐positive/HER2‐negative patients, either without specific mutations or those with *ESR1* mutations [54].

### 3.2 Indicated Population

#### 3.2.1 PI3K Inhibitors

##### 3.2.1.1 Inavolisib

According to FDA and NMPA labeling, inavolisib, in combination with palbociclib and fulvestrant, is indicated for the treatment of HR‐positive, HER2‐negative locally advanced or metastatic breast cancer in adult patients with endocrine‐resistant disease or those experiencing recurrence during or after adjuvant endocrine therapy associated with *PIK3CA* mutations [13, 14, 15, 16].

*Expert Panel Recommendations*: Based on the findings of the INAVO120 study [44], Inavolisib in combination with palbociclib and fulvestrant is recommended for patients with endocrine‐resistant, *PIK3CA*‐mutated, HR‐positive/HER2‐negative locally advanced or metastatic breast cancer. This treatment can be considered during or after the completion of adjunctive endocrine therapy.

##### 3.2.1.2 Alpelisib

According to the FDA drug label, alpelisib is prescribed in combination with fulvestrant for the treatment of HR‐positive, HER2‐negative, PIK3CA‐mutated advanced or metastatic breast cancer [17]. This applies to postmenopausal women or men whose disease has progressed during or after endocrine therapy. At present, alpelisib has not received approval for use in any indications in China.

*Expert Panel Recommendations*: Drawing from the findings of the SOLAR‐1 and BYLieve studies [46, 47, 48, 49], the combination of alpelisib and fulvestrant is recommended as part of endocrine‐based treatment regimens for HR‐positive, HER2‐negative patients with *PIK3CA* mutations who experience disease progression during or after prior treatment.

#### 3.2.2 AKT inhibitors

##### Capivasertib

According to the FDA label, capivasertib, in combination with fulvestrant, is indicated for the treatment of adult patients with locally advanced or metastatic breast cancer who are HR‐positive/HER2‐negative and harbor one or more *PIK3CA*, *AKT1*, or *PTEN* alterations [18]. Eligible patients include those who have experienced disease progression following at least one endocrine‐based therapy during the advanced or metastatic stage or who have relapsed within 12 months of completing adjuvant therapy. Notably, 69% of participants in the CAPItello‐291 trial had previously undergone treatment with a CDK4/6 inhibitor, making it the only major phase III clinical study to evaluate targeted PAM pathway drugs in this specific patient population [51]. Capivasertib is now on the brink of approval for this indication in China.

*Expert Panel Recommendations*: Based on the CAPItello‐291 study [51], capivasertib combined with fulvestrant is recommended for patients with locally advanced or metastatic HR‐positive/HER2‐negative breast cancer who exhibit any gene alterations in *PIK3CA*, *AKT1*, or *PTEN*. This treatment is suitable during or after endocrine therapy in cases of disease recurrence or progression. Additionally, for advanced breast cancer patients with *PIK3CA*, *AKT1*, or *PTEN* alterations who have previously received CDK4/6i, capivasertib in combination with fulvestrant presents a viable treatment option.

#### 3.2.3 mTOR Inhibitors

##### Everolimus

According to the FDA drug label [12] and indications approved by the NMPA, everolimus in combination with exemestane is prescribed for the treatment of postmenopausal patients with HR‐positive/HER2‐negative advanced breast cancer who have not responded to prior therapy with letrozole or anastrozole.

*Expert Panel Recommendations*: Based on the findings from the BOLERO series [55, 56] and the MIRACLE study [57], everolimus combined with exemestane is recommended as a treatment option for HR‐positive/HER2‐negative advanced breast cancer patients whose conditions have progressed following prior endocrine monotherapy or combination therapy.

### 3.3 Drug Information

According to the FDA and NMPA drug labels, information on pharmacokinetics, dosage, and administration of the PI3K inhibitors inavolisib [14, 16] and alpelisib [17], the AKT inhibitor capivasertib [18], and the mTOR inhibitor everolimus [12] is summarized in Table 1.

**Table 1:** Pharmacokinetics, dosage, and administration of inavolisib, alpelisib, capivasertib, and everolimus.

Generic name	Brand name	Dosage form	Manufacturer	Time to maximum plasma concentration (h)	Half‐life (h)	Initial dosage and administration	Dose modification	Route of administration
Inavolisib [14, 16]	Itovebi®	Tablets	Roche	3	15	Swallow tablets whole, 9 mg each time, once daily, continuous administration^a^ ^,^ ^b^	Modify dose according to adverse reactions. Discontinue permanently if patients are unable to tolerate the second dose reduction	Take orally with or without food
Alpelisib [17]	Piqray®	Tablets	Novartis	2−4	8−9	Swallow tablets whole, 300 mg each time (2 tablets, 150 mg each), once daily, continuous administration^a^ ^,^ ^b^ ^,^ ^c^	Modify the dose according to the safety parameters and tolerance of each individual. May discontinue and/or decrease the dosage	Take orally with food
Capivasertib [18]	Truqap®	Tablets	AstraZeneca	1−2	8.3	Swallow tablets whole, 400 mg each time (2 tablets, 200 mg each), orally, twice daily (approximately 12 h apart), for 4 days followed by 3 days off	Modify dose according to adverse reactions. Discontinue permanently if patients are unable to tolerate the second dose reduction	Take orally with or without food
Everolimus [12]	Afinitor®	Tablets	Novartis	1−2	30	Swallow tablets whole, 10 mg each time, once daily, continuous administration^d^ ^,^ ^e^	Modify the dose according to the safety parameters and tolerance of each individual. May discontinue and/or decrease the dosage	Take orally with food or on an empty stomach

^a^ Swallow tablets whole. Do not chew, crush, or split them before swallowing. Do not take tablets that are broken, cracked, or otherwise not intact.

^b^ Advise patients to take inavolisib at approximately the same time each day. If a patient misses a dose, instruct them to take the missed dose as soon as possible, provided it is within 9 h. If more than 9 h have passed, advise the patient to skip the missed dose and take the next dose at the scheduled time. If a patient vomits after taking a dose, instruct them not to take an additional dose on that day but to resume the regular dosing schedule the following day.

^c^ When combined with fulvestrant, the recommended dose of fulvestrant is 500 mg, administered on Days 1, 15, and 29, and once monthly thereafter.

^d^ Swallow tablets whole. Tablets should not be chewed, crushed, or split. Administer them orally at the same time each day. Take everolimus once daily, with or without food, and swallow the tablets with a glass of water.

^e^ Combined with exemestane.

### 3.4 Drug Interactions

Alpelisib, capivasertib, and everolimus are all metabolized by the cytochrome P450 enzyme, CYP3A4. Consequently, co‐administration with strong CYP3A4 inducers can lower blood drug concentrations, diminishing the pharmacological activity of these agents. Conversely, co‐administration with strong CYP3A4 inhibitors can elevate drug concentrations, increasing the risk of toxicity. Therefore, it is recommended to avoid combining these drugs with strong CYP3A4 inducers or inhibitors and to exercise caution when using them alongside moderate inducers or inhibitors of CYP3A4 [12, 17, 18, 58]. Inavolisib acts as an inducer of CYP3A and functions as a time‐dependent inhibitor of CYP3A. It does not inhibit CYP1A2, CYP2B6, CYP2C8, CYP2C9, CYP2C19, or CYP2D6 [14, 16]. Capivasertib may influence the metabolism and clearance of drugs that are CYP3A substrates when used concomitantly [18]. Similarly, alpelisib can reduce the plasma concentrations of drugs that are CYP2C9 substrates, potentially necessitating dose adjustments or close monitoring of drug levels [17].

Transport proteins, such as P‐gp and BCRP, are significant factors influencing drug interactions. Inavolisib acts as a substrate for P‐gp and BCRP but does not inhibit their activity [14, 16]. Similarly, everolimus is a substrate of P‐gp, while alpelisib is a substrate of BCRP. Consequently, everolimus should not be combined with strong inducers or inhibitors of P‐gp, and caution is advised when it is used alongside moderate P‐gp inhibitors. If co‐administration is unavoidable, a dosage reduction should be considered [12, 16, 58]. Additionally, careful evaluation of potential drug interactions is necessary when alpelisib is combined with BCRP inhibitors or inducers [16, 17]. Coadministration of alpelisib with BCRP inhibitors can elevate its concentration, increasing the risk of toxicity; hence, such combinations should be avoided [17].

For guidance on precautions when using PAM pathway inhibitors in combination with other drugs, please refer to Table 2.

**Table 2:** Precautions for combined use of PAM pathway inhibitors with other drugs [12, 14, 16, 17, 18, 56].

Classification	Drug names	Precautions for coadministration of PAM pathway inhibitors with other drugs	PAM pathway inhibitors			
			Inavolisib*	Alpelisib	Capivasertib	Everolimus
Strong CYP3A4 inducers	Including but not limited to phenytoin, carbamazepine, rifampin, rifabutin, rifapentine, and phenobarbital	Strong CYP3A4 inducers may reduce drug concentrations and, therefore, drug efficacy. Avoid coadministration with alpelisib, capivasertib and everolimus. If concomitant use with everolimus cannot be avoided, consider doubling the daily dose of everolimus in increments of 5 mg or less. If the strong CYP3A4 inducer is discontinued, consider a washout period of 3 to 5 days before returning to the previous dose of everolimus.	—	**√**	**√**	**√** a
Moderate CYP3A4 inducers	Including but not limited to lopinavir, lolatinib, bosentan, dabrafenib	Moderate CYP3A4 inducers may reduce drug concentrations and, therefore, drug efficacy. Avoid coadministration with capivasertib.	—	—	**√**	—
Strong CYP3A4 inhibitors	Including but not limited to boceprevir, clarithromycin, posaconazole, ritonavir, voriconazole	Strong CYP3A4 inhibitors may increase drug concentrations and thus the risk of toxicity. Avoid concomitant use with capivasertib and everolimus. If concomitant use with capivasertib cannot be avoided, reduce the capivasertib dosage to 320 mg orally twice daily for 4 days, followed by 3 days off.	—	—	**√**	**√**
Moderate CYP3A4 inhibitors	Including but not limited to fosamprenavir, aprepitant, erythromycin, fluconazole, verapamil, and diltiazem	Moderate CYP3A4 inhibitors may increase drug concentrations and thus the risk of toxicity. If concomitant use with capivasertib cannot be avoided, reduce the capivasertib dosage to 320 mg orally twice daily for 4 days, followed by 3 days off. If concomitant use with everolimus cannot be avoided, reduce the everolimus dose to 2.5 mg/day. If well tolerated, consider increasing the dose from 2.5 to 5 mg/day. When discontinuing a coadministered moderate CYP3A4 inhibitor, allow a wash‐out period of 2‐3 days before increasing the dose of everolimus. After discontinuation, the dose of everolimus should be increased to the same dose before the moderate CYP3A4 inhibitor was added.	—	—	—	**√**
CYP3A substrates	Including but not limited to carbamazepine, cyclosporine, fentanyl, simvastatin	The concentration of drugs eliminated primarily by CYP3A metabolism may be increased when co‐administered with capivasertib. This may result in increased toxicity of these agents, depending on their therapeutic window. Dosage adjustment may be necessary for drugs that are primarily eliminated by CYP3A metabolism and have a narrow therapeutic window.	—	—	**√**	—
CYP2C9 substrates	Including but not limited to warfarin	Coadministration of alpelisib with CYP2C9 substrates may decrease the plasma concentration of these drugs, diminishing their activity. When coadministered with alpelisib, closely monitor the concentration of CYP2C9 substrate.	—	**√**	—	—
Strong P‐gp inhibitors	Including but not limited to ketoconazole, itraconazole and ritonavir	Strong P‐gp inhibitors increase the plasma concentration of everolimus. Avoid concomitant use.	—	—	—	**√**
Moderate P‐gp inhibitors^b^	Including but not limited to amprenavir, fosamprenavir, aprepitant, erythromycin, and diltiazem	Moderate P‐gp inhibitors increase the plasma concentration of everolimus. If concomitant use with everolimus cannot be avoided, reduce the everolimus dose to 2.5 mg/day. If well tolerated, consider increasing the dose from 2.5 to 5 mg/day. When discontinuing a moderate P‐gp inhibitor, allow a wash‐out period of 2–3 days before increasing the dose of everolimus. After discontinuation, the dose of everolimus should be increased to the same dose before the moderate P‐gp inhibitor was added.	—	—	—	**√**
BCRP inhibitors	Including but not limited to cyclosporine	BCRP inhibitors may increase the concentration of alpelisib, increasing the risk of toxicity. Avoid concomitant use. If no alternative regimen is available, monitor adverse effects closely.	—	**√**	—	—

Abbreviations: BCRP, breast cancer resistance protein; P‐gp, P‐glycoprotein.

^a^ St. John's Wort (*Hypericum perforatum*) may unexpectedly decrease exposure to everolimus and should therefore be avoided.

^b^ During treatment, the consumption of grapefruit, grapefruit juice, and other foods known to inhibit cytochrome P450 or P‐glycoprotein (P‐gp) activity should also be avoided.

* The prescribing information does not specify precautions regarding combined use of inavolisib with any particular medications.

### 3.5 Usage in Specific Populations

#### 3.5.1 Pregnant or Lactating Women

Inavolisib, alpelisib, capivasertib, and everolimus have the potential to harm a fetus when administered to pregnant women. Therefore, it is crucial to inform pregnant women of the possible risks these medications pose to fetal health. For individuals of childbearing potential—both women and men—the use of effective contraceptive measures is strongly recommended during treatment and for a designated period after the final dose. The required duration varies by medication: inavolisib (1 week), alpelisib (1 week), capivasertib (4 weeks), and everolimus (8 weeks) [12, 14, 16, 17, 18].

During the treatment of lactating women with inavolisib, alpelisib, capivasertib, or everolimus, breastfed infants may be at risk of experiencing serious adverse events. Consequently, it is recommended that lactating women avoid breastfeeding both during treatment and for a specified period following the final dose. The suggested durations are as follows: inavolisib (1 week), alpelisib (1 week), capivasertib (only during the treatment period), and everolimus (2 weeks) [12, 14, 16, 17, 18].

#### 3.5.2 Patients With Kidney Impairment

Impaired renal function is not expected to influence exposure to everolimus, and no dose adjustment is recommended. However, dosage adjustments for other PAM pathway inhibitors should be guided by the patient's renal function. For patients with mild renal impairment, no dose adjustments are required for inavolisib, alpelisib, or capivasertib. In cases of moderate renal impairment, no dosage modification is needed for alpelisib. However, due to limited data, capivasertib should be used with caution, and the starting dose of inavolisib should be reduced to 6 mg. For severe renal impairment, there is no available experience with inavolisib, alpelisib, or capivasertib; therefore, these medications should be administered with caution [12, 14, 16, 17, 18].

#### 3.5.3 Patients With Liver Dysfunction

Alpelisib does not require dose adjustments in patients with impaired liver function. In contrast, other PAM pathway inhibitors must be adjusted based on the patient's liver function. For patients with mild liver impairment, neither inavolisib nor capivasertib requires dose modification, whereas everolimus should be administered at a reduced dose. In cases of moderate liver impairment, everolimus should be used at a reduced dosage, no clinical experience exists with inavolisib, and because data on capivasertib are limited, it should be used with caution. For patients with severe liver impairment, no data are currently available regarding the use of either inavolisib or capivasertib [12, 14, 16, 17, 18].

#### 3.5.4 Elderly Patients

In the elderly population, no significant differences were observed in the pharmacokinetics, safety, or efficacy of capivasertib. No dose adjustment of inavolisib is required in patients ≥ 65 years, even the rate of dose adjustments or treatment interruptions due to adverse events from inavolisib is elevated in patients ≥ 65 years (79% compared to 68%) [14, 16]. However, when alpelisib is administered to individuals aged 65 years and older, the incidence of grade 3–4 hyperglycemia is notably higher (44% compared to 32% for < 65 years). Furthermore, adverse events from everolimus resulting in treatment discontinuation are more common in patients ≥ 65 years (33% compared to 17%). Consequently, it is strongly recommended that all two drugs—alpelisib and everolimus—be closely monitored for adverse events in the elderly population, with appropriate dosage adjustments implemented as necessary [12, 18].

### 3.6 Clinical Monitoring Indicators and Timing Before and During Drug Administration

#### 3.6.1 PI3K Inhibitors

##### 3.6.1.1 Inavolisib

According to the FDA and NMPA drug label [14, 16], the clinical monitoring indicators, recommended monitoring frequency and associated management guidelines for inavolisib are outlined in Table 3. Both fasting blood glucose (FBG) and HbA1c levels should be assessed before initiating treatment with inavolisib and monitored periodically throughout the course of therapy.

**Table 3:** Recommendations for clinical monitoring indicators and frequency for patients receiving inavolisib [14, 16].

Parameters	Inavolisib
FPG/FBG	Before initiating treatment with inavolisib, test and optimize fasting glucose levels (FPG or FBG).
	After initiating treatment with inavolisib, or in patients who experience hyperglycemia after initiating treatment with inavolisib, monitor or self‐monitor fasting glucose levels once every 3 days for the first week (Days 1 to 7), then once every week for the next 3 weeks (Day 8 to 28), then once every 2 weeks for the next 8 weeks, then once every 4 weeks thereafter as clinically indicated.
HbA1c	Before initiating treatment with inavolisib, test HbA1C levels.
	During treatment with inavolisib, monitor HbA1C every 3 months and as clinically indicated.

Abbreviations: FBG, fasting blood glucose; FPG, fasting plasma glucose; HbA1c, glycosylated hemoglobin.

##### 3.6.1.2 Alpelisib

According to the FDA drug label [17], the clinical monitoring indicators, recommended monitoring frequency, and related management guidelines for alpelisib are outlined in Table 4. Both FPG and HbA1c levels should be assessed before initiating alpelisib treatment and monitored regularly throughout the course of therapy.

**Table 4:** Recommendations for clinical monitoring indicators and frequency for patients receiving alpelisib [17].

Parameters	Alpelisib
FPG	Evaluate and optimize blood glucose before initiating alpelisib treatment.
	After treatment initiation, monitor blood glucose and/or FPG levels at least once a week in the first 2 weeks, and once every four weeks after that. Note: if blood glucose levels are monitored instead of FPG, patients must measure fasting blood glucose levels.
HbA1c	Evaluate HbA1c before initiating alpelisib treatment.
	During alpelisib treatment, monitor HbA1c at least once every 3 months.

Abbreviations: FPG, fasting plasma glucose; HbA1c, glycosylated hemoglobin.

#### 3.6.2 AKT inhibitors

##### 3.6.2.1 Capivasertib

According to the FDA drug label [18], the clinical monitoring indicators for capivasertib align closely with those of alpelisib (Table 4). The recommended frequency for FPG monitoring is at least once every 2 weeks during the first month of treatment, transitioning to at least once a month from the second month onward. For HbA1c, an initial assessment should be conducted before initiating capivasertib therapy, followed by monitoring at 3‐month intervals thereafter.

#### 3.6.3 mTOR inhibitors

##### Everolimus

According to the FDA drug label [12], the clinical monitoring indicators, recommended frequency, and respective management guidelines for everolimus are outlined in Table 5. Key parameters, including kidney function, blood glucose levels, blood lipids, and hematological markers, should be monitored carefully both before and during everolimus treatment.

**Table 5:** Recommendations for clinical monitoring indicators and frequency in patients receiving everolimus [12].

Parameters	Everolimus
Renal function	Elevated blood creatinine levels and proteinuria have been reported in patients receiving everolimus. Monitoring of renal function before initiation of everolimus treatment is recommended, including blood urea nitrogen (BUN), urine protein, or blood creatinine tests, as well as regular tests afterward. For patients with risk factors for further renal impairment, special attention should be paid to their renal function.
Blood glucose and lipid panel	For patients who already have hyperglycemia, hypercholesterolemia, and hypertriglyceridemia, fasting blood glucose and lipid panel should be examined before initiation of everolimus treatment. Regular tests are also recommended after initiation, and appropriate management should be provided. If everolimus is coadministered with other medications that might cause hyperglycemia, more frequent tests are recommended. If possible, ideal blood glucose control should be obtained before treatment initiation.
Hematologic parameters	Decreased hemoglobin, lymphocytes, and platelets have been reported in patients receiving everolimus. A complete blood count should be requested before the initiation of everolimus treatment and regularly afterward.

### 3.7 Management of Adverse Events

#### 3.7.1 General Principles for Managing Adverse Events

The management of adverse events is crucial for ensuring treatment efficacy and improving patients’ quality of life. Since the adverse reaction profiles of various targeted therapies often differ, it is vital to thoroughly understand and monitor the specific adverse events associated with different PAM pathway inhibitors. This enables proactive prevention, vigilant monitoring, and timely implementation of appropriate and effective treatment strategies. In cases of severe adverse events, the use of PAM pathway inhibitors should be paused or discontinued. Multidisciplinary collaboration plays a pivotal role in managing such reactions. Strengthening partnerships with specialists from fields like endocrinology, dermatology, gastroenterology, cardiology, and other relevant disciplines significantly improves the management of adverse events related to PAM pathway inhibitors [59].

#### 3.7.2 Types of Adverse Events, Incidence Rates, and Management Strategies

PAM pathway inhibitors, including inavolisib, alpelisib, capivasertib, and everolimus, demonstrate a manageable overall safety profile. While their adverse reaction spectra are similar, the incidence rates of these reactions vary among the drugs. Common adverse events and their incidence rates for each drug are shown in Table 6.

**Table 6:** Summary of adverse reaction incidence for inavolisib, alpelisib, capivasertib, and everolimus (%).

Adverse reactions	Inavolisib [14, 16]		Alpelisib [17]		Capivasertib [18, 51]		Everolimus [12]	
	All grades	Grade ≥ 3	All grades	Grade ≥ 3	All grades	Grade ≥ 3	All grades	Grade ≥ 3
Hyperglycemia	58.6	5.6	65.0	36.9	16.3	2.3	14.0–42.5	3.0–10.0
Diarrhea	48.1	3.7	58.0	7.0	72.4	9.3	30.0	1.0
Skin rash	25.3	0.0	64.1	22.7	38.0	12.1	27.0–44.0	< 1.0–4.0
Stomatitis	51.2	5.6	30.0	2.5	14.6	2.0	33.8−69.0	2.9−11.0
Non‐infectious pneumonitis	N/A	N/A	1.8	N/A	N/A	N/A	16.0−23.8	4.2
Severe cutaneous reactions	N/A	N/A	1.5	N/A	0.8	N/A	N/A	N/A
Serious hypersensitivity reactions	N/A	N/A	0.7	N/A	0.8	N/A	N/A	N/A
Hyperlipidemia	N/A	N/A	N/A	N/A	N/A	N/A	17.0−57.4	2.0−12.9
Thrombocytopenia	48.1	14.2	N/A	N/A	N/A	N/A	N/A	N/A
Anemia	37.0	6.2	N/A	N/A	10.4	2.0	N/A	N/A
Nausea	27.8	0.6	45.0	2.5	34.6	0.8	26.0	1.0
Fatigue	38.0	1.9	42.0	5.0	20.8	0.6	31.0	5.0
Decreased appetite	24.0	0.0	36.0	0.7	16.6	0.3	N/A	N/A
Vomiting	14.8	0.6	27.0	0.7	20.6	1.7	20.0	2.0

Abbreviation: N/A, not available.

Regarding the adverse events of PAM pathway inhibitors, the expert group categorized and summarized these reactions based on management strategies outlined in clinical studies and drug guidelines. The group recommends adopting individualized management approaches during clinical treatment, tailored to the specific drugs used as well as the type and severity of adverse events. For detailed grading criteria of adverse events and corresponding management recommendations, please refer to Tables 7, 8, 9, 10, 11, 12, 13.

**Table 7:** Grading standards and management recommendations for hyperglycemia with inavolisib [14, 16], alpelisib [17], capivasertib [18], and everolimus [12].

Grade	Definition	Recommendations on dose modification and management
1	ULN < FPG ≤ 160 mg/dL [8.9 mmol/L] or HbA1C > 7%	No need to modify the dose. Consider appropriate oral anti‐diabetic treatment and monitoring.
2	160 < FPG ≤ 250 mg/dL [8.9–13.9 mmol/L]	Inavolisib: withhold until FPG or FBG decreases to ≤ 160 mg/dL (≤ 8.9 mmol/L). Initiate or intensify anti‐hyperglycemic medications. Resume inavolisib at the same dose level. If FPG or FBG persists > 200–250 mg/dL (> 11.1–13.9 mmol/L) for 7 days under appropriate anti‐hyperglycemic treatment, consider a consultation with a healthcare professional experienced in the treatment of hyperglycemia.
		Alpelisib: no dose adjustment is required. Initiate or intensify anti‐hyperglycemic medications. If FPG does not decrease to ≤ 160 mg/dL (≤ 8.9 mmol/L) within 21 days under appropriate anti‐diabetic treatment, reduce the dose by one dose level and follow FPG value‐specific recommendations.
		Capivasertib: withhold until FG decreases to ≤ 160 mg/dL (≤ 8.9 mmol/L). If recovery occurs in ≤ 28 days, resume at the same dose. If recovery occurs in > 28 days, resume at one lower dose.
3	250 < FPG ≤ 500 mg/dL [13.9–27.8 mmol/L]	Inavolisib: withhold and initiate or intensify anti‐hyperglycemic medications. Administer appropriate hydration if required. If FPG or FBG decreases to ≤ 160 mg/dL (≤ 8.9 mmol/L) within 7 days, resume inavolisib at the same dose level. If FPG or FBG decreases to ≤ 160 mg/dL (≤ 8.9 mmol/L) in ≥ 8 days, resume inavolisib at one lower dose level. If FPG or FBG > 250 to 500 mg/dL (> 13.9–27.8 mmol/L) recurs within 30 days, withhold inavolisib until FPG or FBG decreases to ≤ 160 mg/dL (≤ 8.9 mmol/L). Resume inavolisib at one lower dose level.
		Alpelisib: interrupt and initiate or intensify anti‐hyperglycemic medications. Administer appropriate hydration if required. If FPG decreases to ≤ 160 mg/dL (≤ 8.9 mmol/L) within 3 to 5 days under appropriate anti‐diabetic treatment, resume alpelisib at 1 lower dose level. If FPG does not decrease to ≤ 160 mg/dL (≤ 8.9 mmol/L) within 3–5 days under appropriate anti‐diabetic, consult with a physician with expertise in the treatment of hyperglycemia. If FPG does not decrease to ≤ 160 mg/dL (≤ 8.9 mmol/L) within 21 days following appropriate anti‐diabetic treatment, permanently discontinue alpelisib. Capivasertib: withhold until FPG decreases to ≤ 160 mg/dL (≤ 8.9 mmol/L). If recovery occurs in ≤ 28 days, resume capivasertib at one lower dose. If recovery occurs in > 28 days, permanently discontinue capivasertib. Everolimus: withhold until improvement to Grade 0, 1, or 2. Resume at 50% of the previous dose; change to every other day dosing if the reduced dose is lower than the lowest available strength.^a^
4	FPG > 500 mg/dL [27.8 mmol/L]	Inavolisib: withhold and initiate or intensify anti‐hyperglycemic medications. Assess for volume depletion and ketosis and administer appropriate hydration. If FPG or FBG decreases to ≤ 160 mg/dL (≤ 8.9 mmol/L), resume inavolisib at one lower dose level. If FPG or FBG > 500 mg/dL (> 27.8 mmol/L) recurs within 30 days, permanently discontinue inavolisib. Alpelisib: interrupt and initiate or intensify anti‐hyperglycemic medications. Administer appropriate hydration if &QJ10;required. If FPG decreases to ≤ 500 mg/dL (≤ 27.8 mmol/L), follow FPG value‐specific recommendations for Grade 3. If FPG is confirmed at > 500 mg/dL (> 27.8 mmol/L), permanently discontinue treatment. Capivasertib: if FG persists at ≥ 500 mg/dL (> 27.8 mmol/L) after 24 h, permanently discontinue. If FG ≤ 500 mg/dL (≤ 27.8 mmol/L) within 24 h, follow guidance for the relevant grade. Everolimus: terminate therapy^a^

Abbreviations: FBG, fasting blood glucose; FG, fasting glucose; FPG, fasting plasma glucose; HbA1c, glycosylated hemoglobin; ULN, upper limit of normal.

^a^ Recommended dosage modifications for “metabolic events” (e.g., hyperglycemia, dyslipidemia).

**Table 8:** Grading standards for diarrhea and treatment recommendations with inavolisib [14, 16], alpelisib [17], capivasertib [18], and everolimus [12].

**Grade**	**Definition** a	**Recommendations on dose modification and management**
1	Increase of < 4 stools per day over baseline. Mild increase in the frequency of stool, but it is not interfering with daily activities.	No need to modify the dose. Initiate appropriate medication and monitor as clinically indicated.
2	Increase of 4‐6 stools per day over baseline. Interferes with daily activities to some degree.	All: initiate or intensify medical therapy and monitor as clinically indicated. Inavolisib: withhold until recovery to Grade ≤ 1, then resume at the same dose level. For recurrent Grade 2 diarrhea, withhold until recovery to Grade ≤ 1, then resume at one lower dose level. Alpelisib: interrupt until recovery to Grade ≤ 1, then resume at the same dose level. Capivasertib: withhold until recovery to ≤ Grade 1. If recovery occurs in ≤ 28 days, resume at the same dose or one lower dose as clinically indicated. If recovery occurs in > 28 days, resume at one lower dose as clinically indicated. For recurrence, reduce by one lower dose. Everolimus: withhold until improvement to Grade 0 or 1 and resume at the same dose. If toxicity recurs at Grade 2, withhold until improvement to Grade 0 or 1. Resume at 50% of the previous dose; change to every other day dosing if the reduced dose is lower than the lowest available strength.^b^
3	Increase of ≥ 7 stools per day over baseline or incontinence. Requires hospitalization for clinical management. Severely affects normal activities.	All: initiate or intensify medical therapy and monitor as clinically indicated. Inavolisib: withhold until recovery to Grade ≤ 1, then resume at one lower dose level. Alpelisib: interrupt dose until recovery to Grade ≤ 1, then resume at the next lower dose level. Capivasertib: withhold until recovery to ≤ Grade 1. If recovery occurs in ≤ 28 days, resume at the same dose or one lower dose. If recovery occurs in > 28 days, discontinue permanently. Everolimus: withhold until improvement to Grade 0 or 1. Consider resuming at 50% of the previous dose; change to every other day dosing if the reduced dose is lower than the lowest available strength. If toxicity recurs at Grade 3, discontinue permanently.^b^
4	Life‐threatening; urgent intervention indicated	All: initiate or intensify medical therapy and monitor as clinically indicated. Inavolisib: discontinue permanently. Alpelisib: interrupt dose until recovery to Grade ≤ 1, then resume at the next lower dose level. Capivasertib: discontinue permanently. Everolimus: discontinue permanently^b^.

^a^ Based on Common Terminology Criteria for Adverse Events (CTCAE) v5.0.

^b^ Recommended dosage modifications for “other non‐hematologic toxicities.”

**Table 9:** Grading criteria for rash and management recommendations with inavolisib [14, 16], alpelisib [17], capivasertib [18], and everolimus [12].

Grade	Definition^a^	Recommendations on dose modification and management
1	Skin reaction involving < 10% of body surface area (BSA)	Inavolisib: no adjustment required.^b^ Alpelisib: no dose adjustment required. Initiate topical corticosteroid therapy. Consider adding oral antihistamine to manage symptoms.
2	Skin reaction involving 10%–30% of BSA	Inavolisib: considering withholding until recovery to Grade ≤ 1, then resume at the same dose level.^b^ Alpelisib: no dose adjustment required. Initiate or intensify topical corticosteroid and oral antihistaminic therapies. Consider low‐dose systemic corticosteroid therapy. Capivasertib: withhold until recovery to ≤ Grade 1, then resume at the same dose. If persistent or recurrent, reduce by one lower dose. Everolimus: withhold until improvement to Grade 0 or 1 and resume at the same dose. If toxicity recurs at Grade 2, withhold until improvement to Grade 0 or 1. Resume at 50% of the previous dose; change to every other day dosing if the reduced dose is lower than the lowest available strength.^c^
3	Skin reaction involving > 30% of BSA, e.g., severe skin rash unresponsive to medications; daily life affected	Inavolisib: withhold until recovery to Grade ≤ 1, then resume at the same dose level or one lower dose based on clinical evaluation. If a recurrent event, resume at one lower dose level.^b^ Alpelisib: interrupt treatment. Initiate or intensify topical/systemic corticosteroid and oral antihistamine treatment. Once improved to ≤ Grade 1, resume at the same dose level for the first occurrence of rash or at the next lower dose level in case of the second occurrence. Capivasertib: withhold until recovery to ≤ Grade 1. If recovery occurs in ≤ 28 days, resume at the same dose. If recovery occurs in > 28 days, resume at one lower dose. Everolimus: withhold until improvement to Grade 0 or 1. Consider resuming at 50% of the previous dose; change to every other day dosing if the reduced dose is lower than the lowest available strength. If toxicity recurs at Grade 3, discontinue permanently.
4	Severe generalized infection requiring intravenous antibiotics or skin reactions with life‐threatening outcomes, involving any percentage of BSA, e.g., bullous, vesicular, or exfoliative skin diseases	All: discontinue permanently.

^a^ Based on Common Terminology Criteria for Adverse Events (CTCAE) v5.0.

^b^ Recommended dosage modifications for “other adverse reactions.”

^c^ Recommended dosage modifications for “other non‐hematologic toxicities.”

**Table 10:** Common anti‐rash medication regimens. Adapted from [59].

Class	Drug	Route of administration	Recommended dose	Indications
Sunscreen	Broad‐spectrum mineral sunscreen	Topical	SPF ≥15	Face or other exposed areas
Nonsedating anti‐histamines	Cetirizine	Oral	10 mg once daily	Prevention
			10 mg twice daily	Rash of Grade 1 or higher
	Loratadine	Oral	10 mg once daily	Prevention
			10 mg twice daily	Rash of Grade 1 or higher
	Fexofenadine	Oral	180 mg once daily	Prevention
			180 mg twice daily	Rash of Grade 1 or higher
Sedating anti‐histamines	Diphenhydramine	Oral	25–50 mg before bedtime	For burning, tingling, or pruritus
	Hydroxyzine	Oral	25 mg before bedtime	
Corticosteroids	Triamcinolone ointment or cream	Topical	0.1%	Grade 1 or 2 rash
	Fluocinolone acetonide ointment or cream	Topical	0.05%	Grade 1 or 2 rash
	Prednisone	Oral	0.5–1 mg/kg/d or equivalent doses	Grade 3 rash
GABA agonists	Gabapentin	Oral	300 mg three times daily	
	Pregabalin	Oral	50 mg twice daily	

Abbreviation: GABA, γ‐aminobutyric acid.

**Table 11:** Grading standards for stomatitis and management recommendations with inavolisib [14, 16], alpelisib [17], capivasertib [18], and everolimus [12].

Grade	Definition^a^	Recommendations on dose modification and management
1	Asymptomatic or mildly symptomatic, no intervention is required	No need for dose modification. Initiate or intensify appropriate medical therapy (e.g., corticosteroid‐containing mouthwash) as clinically indicated. Gargle with alcohol‐free water or normal saline (0.9%).
2	Moderate pain, swallowing unaffected, texture‐modified food required	Initiate or intensify appropriate medical therapy as clinically indicated. Inavolisib: withhold until recovery to Grade ≤ 1, then resume at the same dose level. If recurrent, resume at one lower dose level. Alpelisib: no dose adjustment is required.^b^ Capivasertib: withhold until recovery to ≤ Grade 1, then resume at the same dose.^c^ Everolimus: withhold until improvement to Grade 0 or 1. Resume at the same dose. If recurs at Grade 2, withhold until improvement to Grade 0 or 1. Resume at 50% of the previous dose; change to every other day dosing if the reduced dose is lower than the lowest available strength.
3	Severe pain, swallowing affected	Initiate or intensify appropriate medical therapy. Inavolisib: withhold until recovery to Grade ≤ 1, then resume at one lower dose level. Alpelisib: interrupt until recovery to Grade < 1, then resume at the next lower dose level.^b^ Capivasertib: withhold until recovery to ≤ Grade 1. If recovery occurs in ≤ 28 days, resume at the same dose. If recovery occurs in > 28 days, resume at one lower dose.^c^ Everolimus: withhold until improvement to Grade 0 or 1. Resume at 50% of the previous dose; change to every other day dosing if the reduced dose is lower than the lowest available strength.
4	With life‐threatening outcomes, emergent intervention is required	All: discontinue permanently.

^a^ Based on Common Terminology Criteria for Adverse Events (CTCAE) v5.0.

^b^ Recommended dosage modifications for “other toxicities (excluding hyperglycemia, rash, and diarrhea).”

^c^ Recommended dosage modifications for “other adverse reactions.”

**Table 12:** Grading criteria for non‐infectious pneumonitis and management recommendations with everolimus [12].

Grade	Definition^a^	Recommendations on dose modification and management
1	Asymptomatic, found in clinical examinations, no intervention required	No need for dose modification. Exclude infectious etiologies and monitor closely.
2	Symptomatic, medication required, activities of daily life unaffected	Consider discontinuation. Exclude infectious etiologies, and consider corticosteroid treatment until symptoms are alleviated to Grade ≤ 1. Withhold until improvement to Grade 0 or 1. Resume at 50% of the previous dose; change to every other day dosing if the reduced dose is lower than the lowest available strength. Discontinue permanently if toxicity does not resolve or improve to Grade 1 within 4 weeks.
3	Severe symptoms, daily life affected, oxygen supplementation required	Withhold until improvement to Grade 0 or 1. Resume at 50% of the previous dose; change to every other day dosing if the reduced dose is lower than the lowest available strength. If toxicity recurs at Grade 3, discontinue permanently.
4	Life‐threatening impairment of respiratory function, emergent intervention required	Terminate therapy

^a^ Based on Common Terminology Criteria for Adverse Events (CTCAE) v5.0.

**Table 13:** Grading criteria and management recommendations for other adverse reactions according to severity for inavolisib [14, 16], capivasertib [18], and everolimus [12].

Grade	Definition	Recommendations on dose modification and management
1	Asymptomatic or mildly symptomatic	No need for dose modification. Initiate appropriate medication and monitoring if necessary.
2	Moderate symptoms	No need for dose modification. Consider discontinuation. Initiate appropriate medication and monitoring if necessary. Inavolisib, Consider withholding, if clinically indicated, until recovery to Grade ≤ 1. Resume at the same dose level. Capivasertib, Withhold until recovery to Grade ≤ 1. Everolimus, no dose adjustment is required if toxicity is tolerable. Withhold until recovery to Grade ≤ 1, then resume at the same dose level, if toxicity is intolerable.
3	Severe symptoms	Inavolisib, (first event) Withhold until recovery to Grade ≤ 1. Resume at the same dose level or one lower dose level based on clinical evaluation. (recurrent) Withhold until recovery to Grade ≤ 1. Resume at one lower dose level. Capivasertib and everolimus: withhold until recovery to Grade ≤ 1, then resume at one lower dose level.
4	Life‐threatening symptoms	Terminate therapy

##### 3.7.2.1 Hyperglycemia

*Expert Panel Recommendations*: It is advised to measure FPG and HbA1c levels before initiating treatment with PAM pathway inhibitors. If necessary, blood glucose levels should be managed in advance. After starting PAM pathway inhibitor therapy, regular monitoring is essential, with adjustments or initiation of antihyperglycemic medications based on clinical indicators. Currently, metformin, which enhances insulin sensitivity to a certain degree, is the preferred treatment option for managing hyperglycemia associated with PAM pathway inhibitors. Additionally, since PAM pathway inhibitors decrease insulin sensitivity, the insulin dosage may need to be increased. If blood glucose levels remain poorly controlled, prompt consultation with an endocrinologist is strongly recommended to develop an appropriate treatment plan.

The grading standards for hyperglycemia, along with corresponding treatment recommendations, are outlined in Table 7.

##### 3.7.2.2 Diarrhea

*Expert Panel Recommendations*: Patients experiencing diarrhea during PAM pathway inhibitor therapy should be advised to manage symptoms by taking loperamide, increasing oral hydration, and informing their healthcare providers. Based on clinical evaluation, appropriate measures—such as dose interruptions, reductions, or discontinuations—should be implemented. For patients with recurrent diarrhea, secondary prophylaxis with loperamide may be considered. The grading criteria for diarrhea, along with corresponding treatment recommendations, are outlined in Table 8.

##### 3.7.2.3 Skin Rash

*Expert Group Recommendations*: The prophylactic use of antihistamines, such as cetirizine (10 mg daily) or loratadine (10 mg daily), is recommended to help reduce the incidence and severity of rashes associated with initiation of PAM pathway inhibitor therapy. Patients should be advised to use mild, fragrance‐free bath soaps and laundry detergents, as well as non‐comedogenic emollients and sunscreens, to maintain skin hydration and health. It is essential to avoid unprotected sun exposure and refrain from using irritating products containing alcohol, salicylic acid, ammonium lactate, urea, or similar substances. Most rashes caused by PAM pathway inhibitors are reversible with appropriate treatment or discontinuation of the therapy. Symptomatic and graded treatments should be administered based on the type of rash and symptom severity. If an infection is present, anti‐infective medication may be required. For all grades of cutaneous drug reactions, irrespective of severity, consultation with a dermatologist is strongly recommended. In cases of persistent rashes or a history of severe (Grade 3) rashes, secondary prophylaxis with ongoing oral antihistamines and/or topical steroids should be considered.

The grading criteria for rashes, along with corresponding treatment recommendations, are outlined in Table 9. Common anti‐rash medication regimens are summarized in Table 10.

##### 3.7.2.4 Stomatitis

*Expert Group Recommendations*: For mild stomatitis, local supportive treatment is generally advised, primarily involving the use of alcohol‐free water or saline gargles along with cold compresses. For moderate to severe stomatitis, topical medications—such as local anesthetics (e.g., lidocaine), glucocorticoids, growth factors, and Kangfuxin solution—are recommended. Additionally, systemic medications, including antibiotics, antifungals, antivirals, analgesics, or other appropriate drugs, may be used in combination as needed. If the condition worsens or requires further intervention, treatment may need to be temporarily paused or discontinued. Equally important is the prevention and long‐term management of stomatitis. Preventative measures include using hormone‐containing mouthwash before treatment, undergoing a thorough dental examination during treatment, and regularly visiting the Stomatology Department for professional cleaning and care. Maintaining good oral hygiene is crucial, as is avoiding irritating foods such as those that are hot, sour, spicy, hard, or brittle.

Refer to Table 11 for the grading criteria of oral inflammation and associated treatment recommendations.

##### 3.7.2.5 Non‐Infectious Pneumonitis

*Expert Panel Recommendations*: Before initiating PAM pathway inhibitors, the following precautions should be observed:

1. Obtain a comprehensive history of the patient's lung‐related conditions.
2. Review recent chest X‐rays and baseline high‐resolution CT scan images, if available.
3. Advise the patient to promptly report any new or worsening respiratory symptoms, such as cough, shortness of breath, or fever.
4. If lung‐related symptoms are present, pulmonary function tests tailored to the patient's specific symptoms are recommended.
5. In cases of diffuse interstitial lung disease, exercise caution when prescribing PAM pathway inhibitors and ensure close monitoring.

The pathological diagnosis of interstitial pneumonia requires a multidisciplinary approach, incorporating clinical symptoms, imaging studies, and pathology. High‐resolution CT scans provide a robust foundation for clinical diagnosis and differentiation. When needed, multidisciplinary consultations should be conducted to ensure accurate assessment and management.

If asymptomatic yet clinically or diagnostically altered pneumonitis is detected during treatment, it warrants closer attention. In cases where symptomatic pneumonitis arises, the use of corticosteroids is advised until symptoms subside to ≤ Grade 1, accompanied by vigilant monitoring. Treatment should be withheld in instances of severe interstitial lung disease or pneumonitis, with reintroduction at a reduced dose only after symptom resolution. For adverse events unresponsive to standard treatment, timely consultation with specialists and multidisciplinary comprehensive management is essential to ensure optimal care.

The grading standards for non‐infectious pneumonia, along with corresponding management recommendations, are outlined in Table 12.

##### 3.7.2.6 Additional Adverse Events

Patients treated with PAM pathway inhibitors also experienced various adverse events, including nausea (26%–45%), fatigue (20.8%–42%), anorexia (16.6%–36%), and vomiting (14.8%–27%). The detailed incidence rates of these adverse events are listed in Table 6 [12, 14, 16, 17, 18]. Generally, no dose adjustment is necessary for Grade 1 or 2 adverse events; instead, appropriate drug therapy and clinical monitoring may be initiated as needed. For Grade 3 adverse events, treatment should be paused until the symptoms resolve to ≤ Grade 1, after which the dose should be reduced in accordance with specific guidelines for PAM pathway drugs. In cases of Grade 4 adverse events, the drug must be permanently discontinued.

For detailed grading standards of other adverse events and corresponding management recommendations, please consult Table 13.

### 3.8 Guidelines for *PI3K/AKT/PTEN* Pathway Key Gene Testing

#### 3.8.1 Clearly Reported Alterations in *PIK3CA*, *AKT1*, and *PTEN* Genes Associated With Efficacy

In the SOLAR‐1 study, the QIAGEN therascreen® *PIK3CA* Rotor‐gene Q (RGQ) polymerase chain reaction (PCR) kit was used to detect 11 common *PIK3CA* mutations, including those located in exons 7, 9, and 20 (C420R, E542K, E545A/D/G/K, Q546E/R, and H1047L/R/Y) [60]. Patients with one or more *PIK3CA* mutations identified in either tissue or plasma samples are eligible for treatment with alpelisib [61]. The PCR kit, along with F1LCDx and F1CDx, has received FDA approval as a companion diagnostic tool for alpelisib [62, 63].

In the INAVO120 study of inavolisib [43], the next‐generation sequencing (NGS)‐based F1LCDx kit was employed to analyze all coding exons of the *PIK3CA* gene and detect a total of 62 specific mutations including the 11 common mutations: H1047D/I/L/N/P/Q/R/T/Y, E545A/D/G/K/L/Q/R/V, E542A/D/G/K/Q/R/V, Q546E/H/K/L/P/R, N345D/HI/K/S/T/Y, C420R, M1043I/T/V, G1049A/C/D/R/S, E453A/D/G/K/Q/V, K111N/R/E, G106A/D/R/S/V, G118D, and R88Q. Patients harboring one or more *PIK3CA* mutations in tissue or plasma samples have shown the potential to benefit from inavolisib‐based therapy. Notably, the F1LCDx kit has been approved by the FDA as a companion diagnostic for inavolisib [62].

In the CAPItello‐291 clinical trial [51], activating mutations in *PIK3CA* and *AKT1*, as well as inactivating alterations in *PTEN*, were identified using NGS‐based F1CDx kits. The detected *PIK3CA* mutations included not only 11 hotspot mutations but also 8 activating mutations—R88Q, N345K, E545Q, Q546K/P, M1043V/I, and G1049R—with an overall population frequency of approximately 5% [64, 65, 66]. Patients carrying activating mutations in *PIK3CA* or *AKT1* or functionally inactivating alterations in *PTEN* may benefit from capivasertib treatment. Functionally inactivated *PTEN* alterations refer to mutations known or predicted to result in loss of function. These specific alterations include missense mutations, nonsense mutations, frameshift mutations, splice site mutations, homozygous deletions, and rearrangements that impair protein function. Notably, F1CDx has received FDA approval as a companion diagnostic tool for capivasertib [62].

#### 3.8.2 Indicated Population for *PIK3CA*, *AKT1*, and *PTEN* Gene Alteration Testing and Specimen Types

According to the Chinese Guidelines for the Diagnosis and Treatment of Breast Cancer [67] and the Guidelines for the Rational Use of Drugs in Breast Cancer [68] issued by the National Health Commission, the era of precision medicine in the clinical diagnosis and treatment of breast cancer has arrived, establishing stringent standards for the rational application of targeted therapies. The NCCN guidelines [45], ABC6/7 guidelines [50], and ASCO guidelines [54], all emphasize the need to test for alterations in the *PIK3CA*, *AKT1*, and *PTEN* genes in patients with HR‐positive/HER2‐negative advanced or metastatic breast cancer. This testing should be conducted via biopsy at the point of disease progression following endocrine therapy. As tissue samples are considered more sensitive for detection, while blood samples better capture the heterogeneity of tumor cell populations, a tissue biopsy should be performed to complement liquid biopsy results if the latter yields a negative outcome [45, 69, 70].

#### 3.8.3 Commonly Employed Gene Testing Methods for *PIK3CA*, *AKT1*, and *PTEN*

Among the molecular pathological detection methods, the most commonly utilized techniques are fluorescence real‐time quantitative PCR (RT‐PCR), microdroplet digital PCR, NGS, and immunohistochemistry (IHC).

Fluorescence RT‐PCR incorporates fluorophores into the PCR process, enabling real‐time monitoring. This method is capable of detecting known mutations and boasts several advantages, including ease of operation, high sensitivity, rapid detection, and cost‐effectiveness.

NGS is a highly advanced parallel sequencing technology capable of simultaneously identifying various major variant types, including single nucleotide variants, small fragment insertions/deletions (indels), gene fusions, large rearrangements, and copy number variants. This technology boasts notable features such as high throughput, exceptional sensitivity, and quantifiability, while also enabling the detection of previously unknown variants, making it increasingly prevalent in clinical oncology applications. However, its complex operational requirements, elevated costs, and extended detection cycles present significant challenges, necessitating more stringent standards for laboratory construction and management.

IHC identifies the expression of specific target proteins by analyzing antigen localization within tissue sections through the immunobinding interaction between antibodies and antigens. This technique offers the advantages of cost efficiency and a quick turnaround time. However, it is not without limitations, as it remains prone to cross‐reactivity and subjective interpretation by observers.

There are notable differences between *PIK3CA* and *AKT1* gene mutations compared to *PTEN* gene alterations. NGS technology is highly effective in detecting alterations in *PIK3CA*, *AKT1*, and *PTEN* genes [51, 62]. Meanwhile, RT‐PCR technology can identify activating mutations in *PIK3CA* and *AKT1*, which are clearly associated with therapeutic efficacy. Additionally, IHC is capable of detecting abnormal protein expression of *PTEN* caused by various types of functional inactivation alterations. In clinical practice, the choice of detection technology should be guided by the specific requirements of genetic testing, the respective strengths and limitations of each method, and the practical considerations of laboratory capabilities.

#### 3.8.4 Compliance Requirements for Molecular Pathology Testing Laboratories

According to the Expert Consensus on Next‐Generation Sequencing Testing in Clinical Molecular Pathology Laboratories, domestic molecular pathology laboratories performing NGS testing must also adhere to the following guidelines: the Guidelines for the Construction of Molecular Pathology Diagnosis Laboratories (Trial), the Guidelines for the Work of Clinical Gene Amplification Testing Laboratories in Medical Institutions, the Quality Assurance Guidelines for Personalized Medicine Testing, the Management Measures for Personalized Medicine Testing Laboratories, the Technical Guidelines for Tumor Personalized Treatment Testing, and the Application Technical Guidelines for Personalized Medicine Testing Using Sequencing Technology (Trial).

During the execution of laboratory test items, the relevant qualification assessments primarily encompass the following six aspects:

1. The tester must hold a “Training Certificate for Laboratory Technicians of Clinical Gene Amplification” issued by the clinical test center.
2. The laboratory must have a clearly defined partition setting.
3. All reagents and instruments must be approved by the NMPA.
4. The quality management system must comply with the requirements of CNAS (China National Accreditation Service for Conformity Assessment) and ISO 15189:2022, “Application Requirements for the Accreditation Criteria for the Quality and Competence of Medical Laboratories” from the American Association of Pathologists, CLIA (Clinical Laboratory Improvement Amendments), or other comparable domestic or international quality system certifications.
5. Laboratory‐developed tests (LDTs) must adhere to the “Regulations for the Supervision and Administration of Medical Devices” as well as applicable national and local LDT pilot program guidelines.
6. The laboratory must actively participate in inter‐laboratory quality evaluations conducted by the Clinical Test Center of the National Health Commission, clinical test centers in various provinces and cities, and organizations such as CAP (College of American Pathologists) and EMQN (European Molecular Genetics Quality Network).

This ensures that all aspects of the laboratory's operations align with stringent regulatory and quality standards.

*Expert Group Recommendations*: It is advised to assess alterations in the *PIK3CA*, *AKT1*, and *PTEN* genes in patients with HR‐positive advanced breast cancer who experience disease progression following endocrine therapy. For optimal accuracy, the detection process should prioritize the most recent histological or blood sample obtained before treatment. If such samples are unavailable, the primary tumor tissue sample should be considered as an alternative.

## 4 Future Outlook

In previously reported clinical data, research on PAM pathway inhibitors has concentrated primarily on their application in first‐line or subsequent treatments for patients with HR‐positive/HER2‐negative advanced or metastatic breast cancer. Abnormal activation of the PAM pathway is not only a potential driver of resistance to endocrine therapy and CDK4/6i but is also closely associated with resistance to anti‐HER2 therapies and chemotherapy. As a result, investigations into the use of PAM pathway inhibitors have increasingly expanded to triple‐negative breast cancer and HER2‐positive breast cancer (Table 14) [71, 72, 73, 74, 75, 76, 77, 78, 79, 80, 81]. In addition, combination strategies for novel PAM pathway inhibitors, including the novel PI3K—mTOR dual inhibitor gedatolisib [82], AKT inhibitor NTQ1062 [83], mTOR inhibitor sapanisertib [84] and vistusertib [85], are also being developed and explored in breast cancer.

**Table 14:** Clinical studies of PAM pathway inhibitors.

Molecular subtype	Patient type	Target	Study	Number of cases	Treatment regimen
HER2 positive	*PIK3CA* mutated advanced breast cancer	PI3K	INAVO122 (NCT05894239) [71]	230^a^	Inavolisib + Phesgo; Placebo + Phesgo (maintenance therapy)
		PI3K	B‐PRECISE‐01 (NCT03767335) [72]	62	MEN1611 + Trastuzumab ± Fulvestrant
HR‐positive/HER2 negative	ER‐positive advanced breast cancer	PI3K	NCT01872260 [73]	255	LEE011 (CDK4/6 inhibitor) + Letrozole; Alpelisib + Letrozole; LEE011 + Letrozole + Alpelisib
	HR‐positive/HER2‐negative, *PIK3CA*‐mutated locally advanced or metastatic breast cancer who have failed standard therapy	PI3K	INAVO121 (NCT05646862) [74]	400^a^	Inavolisib + Fulvestrant; Alpelisib + Fulvestrant
	Endocrine‐Sensitive with PIK3CA‐Mutated, Hormone Receptor‐Positive, HER2‐Negative Advanced Breast Cancer	PI3K	INAVO123 (NCT06790693) [75]	450	Inavolisib + Letrozole + CDK4/6i; Placebo + Letrozole + CDK4/6i
	HR‐positive/HER2‐negative Locally Advanced, Unresectable or Metastatic Breast Cancer	AKT	CAPItello‐292 (NCT04862663) [76]	895	Capivasertib + Fulvestrant and investigator's choice of CDK4/6i; Fulvestrant and investigator's choice of CDK4/6i
	Locally advanced or metastatic HR‐positive/HER2‐negative breast cancer after failure of standard therapy	AKT	NCT04851613 [77]	256^a^	Phase Ib: Afuresertib + Fulvestrant Phase III: Afuresertib + Fulvestrant; Placebo + Fulvestrant
TNBC	Advanced TNBC with *PIK3CA* mutation or *PTEN* loss	PI3K	CBYL719H12301 (NCT04251533, EPIK‐B3) [78, 79]	137	Alpelisib + nab‐Paclitaxel; Placebo + nab‐Paclitaxel (as first‐ or second‐line therapy)
		PI3K	MARIO‐3 NCT03961698) [80, 81]	91	Eganelisib (IPI‐549) + Atezolizumab + nab‐Paclitaxel

Abbreviations: ER, estrogen receptor; HER2, human epidermal growth factor receptor 2; HR, hormone receptor; PAM, phosphoinositide 3‐kinase (PI3K)/protein kinase B (AKT)/mammalian target of rapamycin (mTOR) signaling pathway; TNBC, triple‐negative breast cancer.

a Expected number of participants.

In addition, the selection and optimization of PAM pathway detection methods is important in the precise treatment of breast cancer. However, the application of various detection techniques in clinical practice also faces specific challenges. Therefore, the selection of the PAM pathway detection method should synthesize multiple key factors, including the sensitivity and specificity of detection technology, applicable gene mutation range, resource conditions of clinical practice, and sample characteristics. At the same time, with the development of single‐cell sequencing, multi‐omics integration, and liquid biopsy technology, future clinical applications can further realize full‐cycle and multi‐dimensional dynamic detection and construct an integrated diagnosis and treatment framework covering the gene status and function of the PAM pathway to guide the development of individualized treatment strategies.

## 5 Conclusion

The PAM pathway plays a pivotal role in breast cancer progression, resistance to endocrine therapy, and resistance to CDK4/6 inhibitor therapy, making it a crucial target for breast cancer treatment. Currently, an increasing number of PAM pathway inhibitors have been introduced into clinical practice in China, with many others undergoing research and development. These advancements offer renewed hope for patients with breast cancer both domestically and internationally [5, 11, 37]. This consensus highlights the recent progress and applications of PAM pathway inhibitors over the past 2 years. It further elaborates on drug‐related genetic testing and adverse reaction management, offering specific recommendations for addressing adverse reaction types. At present, PAM pathway inhibitors provide additional treatment options for patients with HR‐positive/HER2‐negative advanced breast cancer following progression on endocrine therapy. Moreover, these inhibitors have demonstrated promising initial efficacy in treating other molecular subtypes of breast cancer and are anticipated to benefit a broader range of patients in the future.

## List of Expert Group Members (All in Alphabetical Order in Chinese Pinyin)

1

**Steering Committee**

Guosheng Ren (Department of Endocrinology and Breast Surgery, The First Affiliated Hospital of Chongqing Medical University); Zhimin Shao (Department of Breast Surgery, Fudan University Shanghai Cancer Center); Jiong Wu (Department of Breast Surgery, Fudan University Shanghai Cancer Center); Binghe Xu (Department of Medical Oncology, National Cancer Center/National Clinical Research Center for Cancer, Cancer Hospital, Chinese Academy of Medical Sciences).

2

**Expert Committee**

Fei Ma (Department of Medical Oncology, National Cancer Center/National Clinical Research Center for Cancer, Cancer Hospital, Chinese Academy of Medical Sciences); Hongnan Mo (Department of Medical Oncology, National Cancer Center/National Clinical Research Center for Cancer, Cancer Hospital, Chinese Academy of Medical Sciences); Jianming Ying (Department of Pathology, Cancer Hospital, Chinese Academy of Medical Sciences; National Cancer Clinical Medical Research Center; National Cancer Center); Yongmei Yin (Department of Medical Oncology, Jiangsu Provincial People's Hospital).

3

**Panelist**

Hong Bu (Department of Pathology, West China Hospital, Sichuan University); Jiuwei Cui (Department of Oncology, the First Hospital of Jilin University); Wei Cui (Department of Clinical Laboratory, Cancer Hospital, Chinese Academy of Medical Sciences, National Cancer Center); Qianjun Chen (Department of Breast Surgery, Guangdong Provincial Hospital of Traditional Chinese Medicine); Wenyan Chen (Department of Medical Oncology, Nanchang People's Hospital); Yiding Chen (Department of Breast Surgery, the Second Affiliated Hospital of Zhejiang University School of Medicine); Zhanhong Chen (Department of Medical Oncology, Zhejiang Cancer Hospital); Huaping Dai (Department of Respiratory and Critical Care Medicine, China‐Japan Friendship Hospital); Lijun Di (Department of Medical Oncology, Peking University Cancer Hospital); Chunfang Hao (Department of Breast Oncology, Tianjin Medical University Cancer Institute & Hospital); Hua Hua (Department of Dermatology, Guang'anmen Hospital, Chinese Academy of Traditional Chinese Medical Sciences); Hai Hu (Department of Breast Oncology, Zhejiang Cancer Hospital); Jian Huang (Department of Oncology, the Second Affiliated Hospital of Zhejiang University School of Medicine); Xichun Hu (Department of Medical Oncology, Fudan University Shanghai Cancer Center); Yuchen Jiao (Department of Medical Oncology, State Key Laboratory of Molecular Oncology, National Cancer Center/National Clinical Research Center for Cancer, Cancer Hospital, Chinese Academy of Medical Sciences); Feng Jin (Department of Breast Surgery, The First Hospital of China Medical University); Hongyuan Li (Department of Endocrinology and Breast Surgery, the First Affiliated Hospital of Chongqing Medical University); Man Li (Department of Medical Oncology, the Second Affiliated Hospital of Dalian Medical University); Huihui Li (Department of Medical Oncology, Shandong Cancer Hospital); Naishi Li (Department of Endocrinology, Peking Union Medical College Hospital); Zhiyong Liang (Department of Pathology, Peking Union Medical College Hospital); Ning Liao (Department of Breast Cancer, Cancer Center, Guangdong Provincial People's Hospital); Qiang Liu (Department of Breast Surgery, Sun Yat‐Sen Memorial Hospital, Sun Yat‐Sen University); Jian Liu (Department of Medical Oncology, Fujian Provincial Cancer Hospital); Wei Li (Department of Medical Oncology, Jiansu Provincial People's Hospital); Yunjiang Liu (Department of Breast Surgery, the Fourth Hospital of Hebei Medical University); Yueping Liu (Department of Pathology, the Fourth Hospital of Hebei Medical University); Zhenzhen Liu (Department of Breast Surgery, Henan Cancer Hospital); Ting Luo (Department of Medical Oncology, West China Hospital, Sichuan University); Fei Ma (Department of Medical Oncology, National Cancer Center/National Clinical Research Center for Cancer, Cancer Hospital, Chinese Academy of Medical Sciences); Hongnan Mo (Department of Medical Oncology, National Cancer Center/National Clinical Research Center for Cancer, Cancer Hospital, Chinese Academy of Medical Sciences); Nengtai Ouyang (Cell and Molecular Diagnosis Center, Sun Yat‐Sen Memorial Hospital, Sun Yat‐Sen University); Quchang Ouyang (Department of Breast Oncology, Hunan Cancer Hospital); Yueyin Pan (Department of Medical Oncology, Anhui Cancer Hospital); Haili Qian (Institute of Cancer Hospital, Chinese Academy of Medical Sciences); Guosheng Ren (Department of Endocrine and Breast Surgery, The First Affiliated Hospital of Chongqing Medical University); Chuangui Song (Department of Breast Surgery, Fujian Medical University); Guohong Song (Department of Medical Oncology, Beijing Cancer Hospital); Tao Sun (Department of Breast Oncology, Liaoning Cancer Hospital); Tong Sun (Yale New Haven Hospital, Surgical Pathologist and Cytopathologist, Bridgeport Hospital, Associate Professor of Pathology, Yale Medical College); Yehui Shi (Department of Breast Oncology, Tianjin Medical University Cancer Institute & Hospital); Yanxia Shi (Department of Medical Oncology, Sun Yat‐sen University Cancer Center); Yuhua Song (Department of Medical Oncology, The Affiliated Hospital of Qingdao University); Zhimin Shao (Department of Breast Surgery, Fudan University Cancer Center); Yuee Teng (Department of Medical Oncology, The First Hospital of China Medical University); Zhongsheng Tong (Department of Breast Oncology, Tianjin Medical University Cancer Institute & Hospital); Haibo Wang (Department of Breast Surgery, the Affiliated Hospital of Qingdao University); Jiong Wu (Department of Breast Surgery, Fudan University Shanghai Cancer Center); Jiayu Wang (Department of Medical Oncology, National Cancer Center/National Clinical Research Center for Cancer, Cancer Hospital, Chinese Academy of Medical Sciences); Kun Wang (Department of Breast Surgery, Guangdong Provincial People's Hospital); Shu Wang (Department of Breast Surgery, Peking University People's Hospital); Shusen Wang (Department of Medical Oncology, Sun Yat‐sen University Cancer Center); Tao Wang (Department of Breast Oncology, The Fifth Medical Center of Chinese PLA General Hospital); Xiaojia Wang (Department of Breast Oncology, Cancer Hospital, Chinese Academy of Sciences/Zhejiang Cancer Hospital); Yongsheng Wang (Department of Breast Surgery, Shandong Cancer Hospital); Zhonghua Wang (Department of Breast Oncology, Fudan University Shanghai Cancer Center); Binghe Xu (Department of Medical Oncology and Clinical Trial Center, National Cancer Center/National Clinical Research Center for Cancer, Cancer Hospital, Chinese Academy of Medical Sciences); Min Yan (Department of Breast Oncology, Henan Cancer Hospital); Hua Yang (Department of Medical Oncology, the Affiliated Hospital of Hebei University); Jin Yang (Department of Medical Oncology, The First Affiliated Hospital of Xi'an Jiaotong University); Jianming Ying (Department of Pathology, National Cancer Center/National Clinical Research Center for Cancer, Cancer Hospital, Chinese Academy of Medical Sciences); Keda Yu (Department of Breast Surgery, Fudan University Shanghai Cancer Center); Wentao Yang (Department of Pathology, Fudan University Shanghai Cancer Center); Yongmei Yin (Department of Medical Oncology, Jiangsu Provincial People's Hospital); Hong Zhang (Department of Pathology, Peking University First Hospital); Hongquan Zhang (Peking University International Cancer Institute); Jian Zhang (Department of Medical Oncology, Fudan University Shanghai Cancer Center); Pin Zhang (Department of Medical Oncology, National Cancer Center/National Clinical Research Center for Cancer, Cancer Hospital, Chinese Academy of Medical Sciences); Qingyuan Zhang (Department of Medical Oncology, Harbin Medical University Cancer Hospital); Yongqiang Zhang (Department of Medical Onchaerbinology, Beijing Hospital), Jiuda Zhao (Breast Center of Affiliated Hospital of Qinghai University), Xuan Zeng (Department of Pathology, Peking Union Medical College Hospital); Xiaoyan Zhou (Department of Pathology, Fudan University Shanghai Cancer Center).

## Author Contributions

**Fei Ma:** conceptualization (equal); data curation (equal); formal analysis (equal); funding acquisition (equal); investigation (equal); methodology (equal); project administration (equal); resources (equal); software (equal); supervision (equal); validation (equal); visualization (equal); writing – original draft (equal); writing – review and editing (equal). **Binghe Xu:** conceptualization (equal); data curation (equal); formal analysis (equal); funding acquisition (equal); investigation (equal); methodology (equal); project administration (equal); resources (equal); software (equal); supervision (equal); validation (equal); visualization (equal); writing – original draft (equal); writing – review and editing (equal).

## Ethics Statement

The authors have nothing to report.

## Consent

The authors have nothing to report.

## Conflicts of Interest

Professor Fei Ma and Professor Binghe Xu are members of the *Cancer Innovation* Editorial Board. To minimize bias, they were excluded from all editorial decision‐making related to the acceptance of this article for publication. The remaining authors declare no conflicts of interest.

## Data Availability

The authors have nothing to report.
